# Cutaneous angiosarcoma: A review of current evidence for treatment with checkpoint inhibitors

**DOI:** 10.3389/fmed.2023.1090168

**Published:** 2023-03-13

**Authors:** Lucy Guan, Marisa Palmeri, Roman Groisberg

**Affiliations:** Department of Medicine, Rutgers Cancer Institute of New Jersey, Robert Wood Johnson Medical School, Rutgers University, New Brunswick, NJ, United States

**Keywords:** cutaneous angiosarcoma, immunotherapy, checkpoint inhibition/blockade, anti-PD-1 immunotherapy, anti-PD-L1

## Abstract

Cutaneous angiosarcoma (cAS) is a rare and aggressive subtype of soft tissue sarcoma with poor prognosis and suboptimal treatment options. Clinical presentation is variable, but cAS often arises from the head and neck. The most widely accepted current approach, surgical excision with adjuvant radiotherapy, is associated with high recurrence rates and can leave patients with profound disfigurement. Chemotherapy and targeted therapy alternatives have had limited success. Thus, there is a significant unmet need to address the absence of durable treatments for advanced and metastatic cAS. Like melanoma and cutaneous squamous cell carcinoma, tumor types with known response to immunotherapy, cAS harbors immune biomarkers, such as tumor mutational burden high (TMB-H), PD-L1 positivity, ultraviolet signature expression, and tertiary lymphoid structures. While data on the use and efficacy of immunotherapy in cAS is limited, the biomarkers suggest a promising advancement in future treatment options. This review aims to summarize and discuss current data from case reports, case series, retrospective studies and clinical trials regarding immunotherapy treatment and outcomes for cAS.

## Introduction

Soft tissue sarcomas (STS), are rare tumors of mesenchymal origin that are made up of over 70 subtypes that vary by tissue of origin, location, histology, immunogenic phenotype, and genomic landscape (1). Angiosarcomas, representing 1 to 2% of all STS, are a heterogeneous and aggressive group of tumors of vascular and lymphatic origin that have the tendency to metastasize to distant visceral sites (2, 3). Cutaneous angiosarcoma (cAS) is the most common form, with the majority of lesions arising from the head and neck (4). Head and neck cAS are associated with advanced age, with a median age of diagnosis of 77, as well as ultraviolet radiation exposure. Overall, the prognosis of patients with cAS is poor, with one analysis indicating a mean 5-year survival rate of 33.5% (3).

For localized cutaneous disease, a combined-modality approach of surgical resection and adjuvant radiotherapy has been the mainstay of treatment despite high recurrence rates. Surgery, however, is contraindicated in many older patients due to age-related comorbidities. Curative radiotherapy has also been reported for local control as local control rates are poor even with resection with wide surgical excision (5). However, due to high distant failure rates and high radiation doses suggested for improved local control (6), this remains a suboptimal treatment modality. For locally advanced or metastatic disease, the first-line standard of care regimen includes cytotoxic chemotherapy, most commonly taxane or anthracycline-based. Radiotherapy can be used as an adjunct. Efficacy of single agent paclitaxel was confirmed in the Phase II ANGIOTAX study of weekly paclitaxel in metastatic or unresectable AS in which 6 of 30 (20.0%) patients had skin or scalp AS. However, median progression free survival (PFS) and median overall survival (OS) were only 4 months and 8 months, respectively (7). More recent studies such as *Roy et al*, which evaluated non-metastatic cAS patients specifically, have shown an overall survival (OS) benefit for concurrent paclitaxel-based chemotherapy and radiotherapy (8). Additionally, radiotherapy with rIL-1 immunotherapy has also been shown to provide improved distant metastasis-free survival rates in patients with angiosarcoma of the scalp (9).

Targeted therapies have also been used in the management of angiosarcomas. Vascular endothelial growth factor (VEGF), which is upregulated in angiosarcoma, can be targeted by tyrosine kinase inhibitors such as pazopanib, regorafenib, sorafenib, and anlotinib as well as monoclonal antibodies such as bevacizumab. Pazopanib which is approved as a second-line agent for the treatment of STS appeared to show a signal of activity (10), but a retrospective study by *Kollar et al* reported limited efficacy. Of the 40 AS patients, which included 15 (37.%) patients with cAS, there was a response rate of 20%, median PFS of 3 months, and median OS of 9.9 months (11). In a phase II trial of anlotinib, a multi-kinase inhibitor, 4 patients had cAS and none of them had objective responses (12). Similarly underwhelming results were seen for regorafenib (13), sorafenib (14), and bevacizumab (15). Alternatives are needed to attain more significant and durable treatment responses.

There has been increased interest in immune regulation as a potential therapeutic avenue in sarcoma treatment, prompting investigation of whether anti-programmed cell death protein 1 (PD-1) or ligand (PD-L1) immune checkpoint inhibitors may have a role in treatment. Suggested biomarkers of response to immunotherapy include tumor infiltrating lymphocytes (TILs), PD-L1 expression, microsatellite instability, immunogenic genomic profile, UV signature, inflamed hypermutated tumors, and tertiary lymphoid structures (16). Tumor mutational burden (TMB) high (≥10 mutations/Megabase (Muts/Mb)) and microsatellite instability high (MSI-H) are biomarkers for which pembrolizumab (17), an anti-PD-1 agent, is approved in the tumor-agnostic setting (18, 19). Furthermore, a study by *Honda et al* investigated the association between PD-1/PD-L1 expression and cAS prognosis. Among 106 immunohistochemically studied cAS cases, 30.2% of patients’ samples were positive for PD-L1, and 17.9% showed high infiltration of PD-1 positive cells. Univariate analyses revealed a significant relationship between high infiltration of PD-1 positive cells with tumor site PD-L1 expression and favorable survival in stage 1 patients (*p* = 0.014). Regression analyses also revealed that patients with high infiltration of PD-1-positive cells with tumor site PD-L1 expression had an increased likelihood of favorable survival, after adjustment with possible confounders (hazard ratio = 0.38, *p* = 0.01, 95%CI: 0.16–0.86).

Certain biomarkers known to be positive in cAS suggest potential for response to immunotherapy. For instance, in head and neck cAS the median TMB is 20 Muts/Mb (20) and in an analysis of 143 angiosarcomas by *Espejio et al* PD-L1 positivity was seen in 33% of head and neck cAS. Head and neck cAS also harbors similarities to known immune checkpoint inhibitor (ICI) responsive tumor types, such as melanoma and cutaneous squamous cell carcinoma (21). One similarity includes the presence of ultraviolet (UV) mutational signatures, defined as a high number of genomic variations caused by demethylation of cPG islands, which is associated with response to anti-PD-1 agents (22). In an evaluation by *Chan et al*, 9 of 18 head and neck cAS patients had UV mutational signatures (23, 24). Another similarity to melanoma and cSCC includes the predominant immune rich microenvironment of cAS, specifically of the head and neck region, with the presence of high levels of CD8+ TILs (24–28).

Given the lack of durable treatment options for advanced or metastatic cAS, further treatments beyond chemotherapy and targeted therapy are necessary to address this significant unmet need. Data, albeit promising, on the use and efficacy of immunotherapy in cAS is limited. The present review aims to summarize and discuss current data from case reports, case series, retrospective studies and clinical trials regarding immunotherapy treatment and outcomes for cAS.

### Clinical studies supporting use of immunotherapy in cAS

There are numerous case reports, case series, retrospective studies and clinical trials that report promising responses to immune checkpoint inhibition in patients with cAS (Table 1). The results are summarized as follows:

**Table 1:** Summary of case reports, case series, and completed clinical trials.

Report	Type	#cAS (%)	ORR	Duration of response	PFS	OS	Notes
Sindu et al. (29)	Case report	1 (100)	n/a	Ongoing	n/a	n/a	
Hamacher et al. (30)	Case report	1 (100)	n/a	Ongoing	n/a	n/a	
Florou et al. (31)	Case series	5 (100)	80%	Ongoing in 4 patients, 14 weeks in 1 patient	n/a	n/a	
Rosenbaum et al. (32)	Retrospective study	15 (42.9)	n/a	n/a	cAS, median: 17.9 weeks	cAS: Not reached	
Phase II SWOG S1609 (DART) trial (33)	Study	Head and neck cAS: 5 (31.3)	Head and neck cAS: 60% Overall: 25%	n/a	6 m PFS rate: 38% (95% Cl 20 to 70%)	Overall: Not reached	3 of 5 head and neck cAS patients had a confirmed objective response to treatment. 2 radiation induced breast cAS patients were excluded from this table.
Phase II study of durvalumab plus tremelimumab (NCT02815995) (34)	Study	1 (1.8)	Overall: 12%	n/a	Overall, median: 12.2 weeks	Overall, median: 93.9 weeks	The 1 cAS patient had a partial response and was the only responder of 5 AS patients.
Phase II study of TVEC and pembrolizumab (NCT03069378) (35)	Study	3 (15)	Overall: 35%	Overall: 56.1 weeks	Overall, median: 17.1 weeks	Overall, disease specific survival: 74.6 weeks	Partial response in 2 cAS patients. Both completed 52 weeks of study therapy.

## Case reports

A case report published by *Sindhu et al* describes a 63-year-old Caucasian man with refractory cAS of the nose who was treated with pembrolizumab after surgical resection and adjuvant chemotherapy with nab-paclitaxel and evofosfamide was insufficient to control his disease (29). Significant disease progression led to development of multiple new hepatic lesions, a jaw mass, and a right tongue mass. Tumor tissue staining revealed positive PD-L1 expression, as measured by >5% of tumor cells staining positive. Off-label treatment with pembrolizumab dosed at 2 mg/kg every 21 days was initiated with concurrent radical excision of jaw soft tissue angiosarcoma. Restaging CT scans during treatment revealed a significant response of the liver lesion. Additional body scans after 1 year revealed further reduction and no new disease.

Hamacher et al. describe a 74-year-old man with left retroauricular cAS who underwent primary resection, radiotherapy to localized disease, and surgical re-resection after locoregional recurrence, including lymph node metastases (30). After complete disappearance of local lesions following 10 cycles of liposomal pegylated doxorubicin (30 to 35 mg/m^2^ once every 28 days), he developed multifocal progression in the left submandibular and right temporoparietal region. He then started on second-line chemotherapy of paclitaxel 80 mg/m^2^ once per week resulting in partial response of the cutaneous lesions lasting for 4 months. Due to peripheral neuropathy induced by chemotherapy, he then switched to trabectedin 1.5 mg/m^2^, which was discontinued due to poor tolerance. During this time, the patient’s cutaneous lesions progressed significantly leading to ulcerations with constant bleeding and requiring analgesics. Fourth-line therapy with pazopanib 800 mg/day was initiated; however, there was continued progression and need for weekly red blood cell transfusions. A histopathological examination of the biopsy taken at recurrence revealed expression of PD-L1 on sarcoma cells of 10% of the sections, prompting the patient’s care team to initiate treatment with pembrolizumab 2 mg/kg once every 21 days. Within 3 weeks, the patient’s lesions had improved significantly; ulcerations had stopped bleeding and as they continued to heal, blood transfusions and analgesic use were discontinued. A good clinical response of all sarcoma lesions was noted after 5 cycles of pembrolizumab, and after an additional 5 cycles, ulcerations had healed completely. The patient had also tolerated pembrolizumab well without clinically relevant toxicities.

### Case series and retrospective study

A case series by Florou et al. describes seven total patients, including five patients with chemotherapy-refractory cAS of the head and neck (31). All patients had received prior systemic chemotherapy and, following progression, received 5 to 14 doses of ICI. After 12 weeks of treatment with AGEN1884, an experimental monoclonal IgG1 antibody targeting CTLA-4, one patient with locally advanced cAS of the face had an ongoing complete response, marking the first reported complete response in cAS to anti-CTLA-4 monotherapy. TMB was surprisingly low at only 0.09 muts/mb, which further highlights the need to explore additional biomarkers. A second patient with cAS of the nose progressed through anti-CTLA-4 monotherapy. Another patient with metastatic cAS with lymph node and bone involvement was treated with pembrolizumab with a partial response for 14 weeks followed by ipilimumab/nivolumab with ongoing partial response. This patient had TMB-H of 15 muts/mb. The remaining two patients had multifocal cAS with scalp involvement and were treated with pembrolizumab, resulting in ongoing partial response. One of these patients had TMB of 12 muts/mb, while the other lacked sufficient archived tumor tissue for analysis.

A retrospective analysis of 35 patients with AS, of whom 15 (42.9%) had cAS, treated with ICI-based therapy aimed to clarify patterns of response and identify prognostic or predictive biomarkers (32). The study performed a retrospective analysis of patients treated with various ICI regimens and investigated correlations between clinical benefit, defined as PFS ≥16 weeks, and various clinical characteristics, results of exome and transcriptome sequencing, and immunohistochemical analyses. ICI regimens were categorized as follows: ICI monotherapy (anti-PD-1 or anti-PD-1 therapy alone), ICI combination therapy (anti-CTLA-4 with anti-PD-1 therapy), and ICI plus other (anti-PD-1 or anti-PD-L1 agent plus a novel immunomodulatory therapy). The median PFS was 11.9 (95%CI 7.4 to 31.9) weeks, and about 40% of all patients had PFS of ≥16 weeks. The study found that patients who received ICI in combination with another novel immune modulator had longer survival rates than patients who received either ipilimumab plus nivolumab or ICI monotherapy. This finding further suggests that novel combinatorial therapies may be more effective in improving outcomes, compared to ICI monotherapy.

### Combination immunotherapy

Building upon these cases, series, and retrospective studies, the following trials provide further insight on the utility of combination therapy.

## Anti-PD-L1 and anti-CTLA-4 combination therapy

The following two trials have explored the efficacy of combining anti-PD-L1 and anti-CTLA-4 therapies:

An angiosarcoma cohort (cohort 51) was added to the multicenter phase II SWOG S1609 (DART) trial. This was the first prospective trial of immunotherapy in AS, examining dual anti-CTLA-4 and anti-PD-1 blockade with ipilimumab and nivolumab in metastatic or unresectable AS (33). Sixteen AS patients, who had a median age of 68 years, were enrolled. Genomic characterization as part of routine medical care was only available for eight patients, which revealed that all eight patients had at least 2 deleterious genomic alterations, with no two patients having the same set of alterations. One of seven patients whose TMB was analyzed showed high TMB of 24 muts/mb; all others ranged from 0 to 8.4 muts/mb. Of the three patients with available PDL-1 immunohistochemistry, PDL-1 tumor proportion score (TPS) was 0% (arm), 30% (skin of face), and 50% (scalp). Nine patients had cutaneous primary tumors, among which five had primary tumors arising from the face or scalp and four had primary tumors of other sites, including two with radiation-associated cutaneous breast tumors. The remaining seven had non-cutaneous primary tumors. Patients received intravenously nivolumab 240 mg every 2 weeks and ipilimumab 1 mg/kg every 6 weeks. Overall, the objective response rate (ORR) was 25% (95% CI: 9 to 45%), 6-month PFS was 38% (95% CI: 20 to 71%), and 12.5% (2 of 16) experienced grade 3 to 4 serious adverse events. The most common adverse events were ALT/AST elevation, diarrhea, hypothyroidism, pneumonitis, pruritis, and rash. Three of the five patients (60%) with cAS of the head and neck had partial responses to therapy. One of these patients had a high TMB of 24 muts/mb (PD-L1 status was unavailable), while another had strong PD-L1 expression at 30% and a TMB of 8.4 muts/mb.

A single center, phase II multi-arm study evaluated the combination of anti-PD-L1 durvalumab and anti-CTLA-4 tremelimumab in 57 patients with various advanced or metastatic sarcoma subtypes (34). The cohort had a median (range) age of 48 (22–77) and a median (range) of 2 (0–6) prior lines of therapy. The study yielded mOS of 21.6 months (95%CI: 12.3–30.9), and mPFS of 2.8 months (95%CI: 1.8–6.4). Fourteen (24.6%) patients experienced grade > 3 related adverse events. Five patients in the study cohort had AS, including one cAS patient who achieved partial response. The study also found higher TIL immune scores to be associated with clinical benefit.

## Anti-PD-L1 and T-VEC combination therapy

Combination pembrolizumab and oncolytic virus therapy Talimogen Laherparapvec (T-VEC) was assessed in an ongoing single center, open-label phase II study that enrolled 20 patients with metastatic and advanced sarcoma (35). The safety and efficacy of this combination therapy was previously demonstrated in patients with melanoma, thus supporting potential similar outcomes in cAS patients (36, 37). Overall, ORR was 35% (7 of 20) with all objective responders achieving partial responses. Of patients with recurrent locally advanced disease, the ORR was 75%. Where tissue samples were available for analysis, positive PD-L1 expression was detected in 83% of responders and 38% of non-responders. There were three cAS patients enrolled on study. Among the responders, two patients had recurrent locally advanced cAS and completed 52 weeks of treatment on study. One patient received 3 prior lines of chemotherapy as well as prior immunotherapy (TIGIT ab and atezolizumab) while the other received one prior line of chemotherapy but no prior immunotherapy. The ongoing trial plans to report further evaluation of T-VEC in combination with pembrolizumab.

### Ongoing trials

Several ongoing efforts may further elucidate the utility of immunotherapy in patients with cutaneous angiosarcoma. Ongoing clinical trials in the U.S. are detailed in Table 2. Additionally, an ongoing phase II study in Japan is assessing response rates to nivolumab for patients with unresectable or metastatic cAS refractory to first-line paclitaxel (38).

**Table 2:** Ongoing clinical trials.

NCT	Trial name	Angiosarcoma	Enrollment status	Preliminary data
NCT04784247	Lenvatinib and Pembrolizumab in People with advanced soft tissue Sarcoma	Includes experimental arm for vascular sarcomas (including angiosarcoma and epithelioid hemangioendothelioma)	Recruiting	Ongoing
NCT04668300	Oleclumab and Durvalumab for the treatment of recurrent, refractory, or metastatic sarcoma (DOSa)	Includes patients with metastatic angiosarcoma, recurrent angiosarcoma	Recruiting (estimated enrollment of 75 participants)	Ongoing
NCT03069378	A study of Talimogene Laherparepvec (T-VEC) in Combination with Pembrolizumab in Patients with metastatic and/or locally advanced Sarcoma	Includes patients with sarcoma, epithelioid sarcoma, and cutaneous angiosarcoma	Recruiting (estimated enrollment of 50 patients)	See above
NCT04339738	Testing the addition of Nivolumab to Chemotherapy in Treatment of Soft Tissue Sarcoma	Skin angiosarcoma, skin radiation-related angiosarcoma, visceral angiosarcoma	Recruiting (estimated enrollment of 90 participants)	Ongoing

## Discussion

Current evidence of immune checkpoint blockade treatment in patients with cAS highlights the exciting future of cAS treatment. The overall positive results in cAS, particularly head and neck, are consistent with its known biomarkers of response to ICI such as TMB-High, PD-L1 positivity, UV mutational signature, and presence of TILs in the tumor microenvironment. A number of these immune biomarkers are predictive of respond to immunotherapy, notably those also found in patients with melanoma and squamous cell carcinoma, suggesting promising future directions for cAS treatment. Large phase II and phase III trials of ICIs in cAS are warranted, especially those with longer follow-up times to better evaluate recurrence and overall survival. Continued challenges include the rarity of AS as well as the heterogeneity within the subtypes; this makes it difficult to attain sufficient cAS patients within larger AS and sarcoma trials. To further evaluate AS and other sarcomas that may respond to ICIs, biomarker-based criteria should be considered for clinical trials. Challenges regarding this technique, however, include the wide variety of biomarkers to select from, and that there are multiple interacting factors that influence response. Additionally, precision biomarker-restricted therapy will inevitably encounter practical challenges associated with the high cost of research and development of companion diagnostics.

## Author contributions

LG and MP performed literature review, wrote original draft and all edits, created tables and were involved in manuscript from conception. RG provided guidance and direction, literature review, and review of manuscript as well as formulation of background and discussion. All authors contributed to the article and approved the submitted version.

## Conflict of interest

RG is an employee of Merck, Sharp & Dohme LLC, Rahway, NJ, United States.

The remaining authors declare that the research was conducted in the absence of any commercial or financial relationships that could be construed as a potential conflict of interest.

## Publisher’s note

All claims expressed in this article are solely those of the authors and do not necessarily represent those of their affiliated organizations, or those of the publisher, the editors and the reviewers. Any product that may be evaluated in this article, or claim that may be made by its manufacturer, is not guaranteed or endorsed by the publisher.
